# Some Questions and Answers About the Role of *Hox* Temporal Collinearity in Vertebrate Axial Patterning

**DOI:** 10.3389/fcell.2019.00257

**Published:** 2019-11-29

**Authors:** Antony J. Durston

**Affiliations:** Institute of Biology Leiden, Leiden University, Leiden, Netherlands

**Keywords:** *Hox* genes, time space translation, axial patterning, gastrulation, stem cells, *Hox-Hox* interactions, BMP-anti BMP

## Abstract

The vertebrate anterior-posterior (A-P = craniocaudal) axis is evidently made by a timing mechanism. Evidence has accumulated that tentatively identifies the A-P timer as being or involving *Hox* temporal collinearity (TC). Here, I focus on the two current competing models based on this premise. Common features and points of dissent are examined and a common model is distilled from what remains. This is an attempt to make sense of the literature.

## Introduction

**FIGURE 1:**
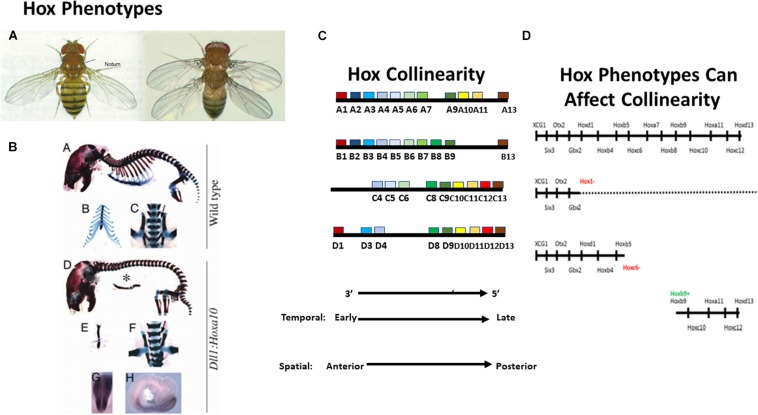
Introducing the *Hox* genes. The *Hox* genes specify different levels along the anterior-posterior (A-P) axis. Their function is obvious from gain and loss of function phenotypes. **(A)** A *Hox* phenotype. Loss of function for Ubx (Hox7 specificity) in Drosophila. The T3 thoracic segment is converted to T2 (having wings instead of halteres Lewis, 1978). **(B)** Another *Hox* phenotype. Gain of function (GOF) (ectopic expression) for Hoxa10 in mouse. The thoracic part of the vertebral column, which bears ribs, is converted to lumbar (abdominal) vertebral column, which does not bear ribs (Carapuço et al., 2005). These findings illustrate that gain and loss of function for particular *Hox* genes converts one part of the A-P axis to another. **(C)** *Hox* collinearity. Vertebrate *Hox* genes groups of colored blocks) are found in four incompletely homologous chromosomal clusters on different chromosomes. Each *Hox* cluster shows temporal collinearity (the *Hox* genes are expressed sequentially in time, from 3′ to 5′ during early development) and spatial collinearity (the *Hox* genes come to be expressed 3′–5′ in a spatial sequence along the early A-P body axis). Invertebrates have only one *Hox* cluster which is generally orthologous to each of the four vertebrate clusters (not shown). **(D)** Vertebrate *Hox* gain and loss of function phenotypes can affect collinearity. Above: Vertebrate Wt. *Hox* sequence. Next: Loss of function for the Xenopus Hox1 paralog group (all 3 Hox1 genes knocked down using morpholinos) Expression of all Hox1 genes and of all more posterior paralog groups is deleted or strongly compromised (McNulty et al., 2005). Next: Loss of function for Hoxc6 (morpholino). Expression of all Hox6 genes and of all more posterior paralog groups is deleted or strongly compromised (Zhu et al., 2017b). Next: GOF for Hoxb9 (ectopic expression by mRNA injection into a dorsalised hoxless embryo). A partial axis is generated, starting at Hoxb4 and proceeding posteriorward. Equivalent results were obtained using ectopic iexpression of: Hoxd1, Hoxa7, and Hoxb4 (Zhu et al., 2017a). These results indicate that Xenopus *Hox* genes interact during A-P axis formation. See text.

Much evidence points to the conclusion that the vertebrate A-P axis is made by a timing mechanism (Nieuwkoop, 1952; Eyal Giladi, 1954; Selleck and Stern, 1991; Collier et al., 2000; Gamse and Sive, 2000, 2001; Vasiliauskas and Stern, 2001; Wacker et al., 2004; Stern et al., 2006; Deschamps and Duboule, 2017). The current evidence tentatively identifies the vertebrate axial patterning timer as being or involving *Hox* temporal collinearity (TC) (i.e., the correspondence of the temporal sequence of *Hox* gene expression during early development with the genomic sequence of *Hox* genes in each cluster). This evidence is presented below. I measure the evidence here against the two current models based on this premise. The following analysis examines, answers and draws conclusions from some of the questions raised. I center it around comparing and contrasting the two recent models [Durston and Zhu, 2015; Durston, 2015, 2019c (dur), Deschamps and Duboule, 2017 (dedu)]. *Conclusion: An analysis of the important facts around *Hox* collinearity and timing in axial patterning is required because this is a complex subject where there is still much to be understood and there are conflicting ideas that need to be resolved. This article strives to make sense of the literature. In addition to the analysis below, *Hox* genes and their collinearity are introduced in Figure 1, the main points of each of the two models are memorized in Table 1 and the abbreviations and terminology used in this paper are listed and defined in Table 2. These features are intended to make this paper accessible to non-specialists.*

**TABLE 1:** Comparison of two main models for the role of *Hox* temporal collinearity in vertebrate A-P axial patterning.

**Feature**	**Deschamps/Duboule model (dedu)**	**Durston model (dur)**
*Hox* temporal collinearity	Yes	Yes
Temporal collinearity leads to spatial collinearity	Yes	Yes
Collinear opening of *Hox* chromatin	Yes	Yes
Intercellular coordination of collinearity by BMP-anti BMP	No	Yes
Intercellular coordination of collinearity by Wnt, FGF-cdx	Yes	Yes
*Hox* PI and A interactions	No	Yes
PP/PD	Yes. PP	Yes. PD
Activation = transformation	No (but this exists)	Yes
NMP’s	Yes	No (but they exist)

**TABLE 2:** Alphabetical list and definitions of abbreviations and terminology.

A	Anterior	Front or upper (human) end of the A-P axis
	Activation-transformation	The classical cell interaction mechanism whereby *Hox* information is transferred from mesoderm to neurectoderm during A-P axial patterning. Discovered in Amphibia (anamniote). Confirmed in chicken (amniote)
Au	Autoregulation	Induction of the expression of a *Hox* gene by itself or a paralog
NMP	Neural-mesodermal precursor	A precursor cell that gives rise to (axial) neural as well as mesodermal precursors. From mouse embryology. A novel way for mesoderm and neurectoderm to share *Hox* information
P	Posterior	Back or lower (human) end of the A-P axis
PD	Posterior dominance	Repression of the expression or inhibition of the function of a more 3′ anterior *Hox* gene by a more 5′ posterior *Hox* gene, leading to functional dominance
PI	Posterior induction	Induction of the expression of a more 5′ posterior *Hox* gene by a more 3′ or anterior *Hox* gene. Generally, this applies to closely neighboring genes but may act as a cascade
PP	Posterior prevalence	A term coined by D. Duboule (1991) Similar to PD except that this is purely functional (no regulation of *Hox* expression)
SC	Spatial collinearity	Spatial sequence of the expression of *Hox* genes that matches their genomic sequence in a *Hox* cluster. Seen for example along the main body axis in most bilaterian embryos
TC	Temporal collinearity	Temporal sequence of the expression of *Hox* genes matching their genomic sequence. Seen in early vertebrate embryos before spatial collinearity and leading to it
TST	Time space translation	The process whereby temporal collinearity generates spatial collinearity

## Does *Hox* Temporal Collinearity Exist?

The two recent models depend on *Hox* TC mediating a developmental timer (also called a “*Hox* clock”). However, recent publications have also denied the existence of *Hox* TC (Kondo et al., 2017, 2019). These papers primarily used RNA seq. analysis and RT-qPCR detection of pre-spliced RNA in whole embryos. This denial has been disputed (Durston, 2019a,b). Does TC exist and how does it work (via expression of a full collinear sequence of *Hox* genes or by approximately synchronous expression of temporally sequential blocks of *Hox* genes)? The clearest evidence on this comes from using *in situ* hybridization to examine tissue specific spatiotemporal expression of *Hox* genes. This is currently the best available method because it allows different locations to be distinguished and therefore avoids confusion inherent in whole embryo analysis due to superposing *Hox* expression in different tissues and at different locations. Precisely directed single cell RNA seq. analysis (Fabre et al., 2018) or fluorescence-activated cell sorting may possibly provide a useful extension in future. The *in situ* hybridization studies, in frog, chicken and mouse embryos, show almost perfectly sequential temporally collinear expression (Izpisúa-Belmonte et al., 1991; Gaunt and Strachan, 1996; Wacker et al., 2004; Iimura and Pourquie, 2006; Denans et al., 2015; Gouveia et al., 2015; Moreau et al., 2018). There is only occasional synchrony in expression (e.g., between *Hoxb8*, *Hoxb9* in Gouveia): not expression in large synchronized blocks. This expression timing generates a nested “Russian Doll” expression pattern, with the individual *Hox* patterns expanding from a common initiation point. *Conclusion: Hox TC does exist and it works via almost fully sequential expression of a collinear sequence of Hox genes (dedu, dur).*

## The Models Propose That *Hox* Temporal Collinearity Leads to *Hox* Spatial Collinearity and Axial Patterning. Does This Occur?

Temporal collinearity leading to spatial collinearity (SC) was first proposed by Duboule and his collaborators (Dollé et al., 1989; Duboule, 1994). Duboule and colleagues made important contributions to the field (cf. Tschopp et al., 2009; Deschamps and Duboule, 2017) and the observations that TC precedes SC in development (Gaunt and Strachan, 1996; Deschamps and Duboule, 2017) and that both can arguably be manipulated coordinately by chromosomal rearrangements (Tarchini and Duboule, 2006; Tschopp et al., 2009) both point to a possible TC–SC link but the definitive evidence that TC leads directionally to SC remained elusive. That evidence and insight into the nature of the connection was finally delivered by Wacker et al. (2004), who showed that *Xenopus* temporal and spatial collinearities can be manipulated, are interchangeable (from TC to SC) and are regulated by *BMP*/anti *BMP*. *BMP* rich ventralised gastrula embryos show only temporal (not spatial) collinearity, reflecting the TC normally found in the embryo’s ventrolateral non-organiser (NOM) mesoderm. If they are challenged with anti-*BMP* (*noggin*) solution injected into the blastocoel: (pulse signal) or an anti-*BMP* producing organizer [introducing a continuous signal (step)], they generate parts of the spatially collinear *Hox* axial pattern, the part generated depending on the time of the challenge and its nature. Early challenges generate or initiate at anterior parts (one to a few sequential anterior zones: early *noggin* pulse or axial sequence starting at an anterior level: early implanted organizer). Sequentially later challenges generate more posterior zones or initiate at sequentially more posterior levels in the axis.

Conclusion: Hox TC leads to Hox SC and axial patterning (dedu, dur). The evidence for this comes from BMP-anti BMP regulation of collinearity (dur). The connection between BMP-anti BMP [a dorsoventral (D-V) patterning antagonism] and A-P patterning reflects the famous connection between vertebrate D-V and A-P patterning (Lane and Sheets, 2002).

## Is *Bmp*/Anti *Bmp* of General Importance?

The above findings showing *BMP*/anti *BMP* as general *Hox* regulating factors were made in *Xenopus*. Genesis of a sequence of specific A-P levels in the axial pattern by specifically timed anti *BMP* signals has also been shown in chicken and zebrafish embryos. In chicken, this concerned induction of an A-P sequence of *Hox* genes by *noggin* in the posterior primitive streak (Dias et al., 2014). In zebrafish, this timed sequence (induced by timed heat shock induction of TS-chordin) starts, interestingly, anteriorly in the non-*Hox* anterior head part of the axis (Tucker et al., 2008; Hashiguchi and Mullins, 2013). In *Xenopus*, where the zebrafish expt. was repeated and expanded, it continues even further into the most anterior EAD (extreme anterior domain) (Jacox et al., 2014; Zhu et al., 2019). The fact of an anti-*BMP* dependent A-P time sequence of stabilized induced states implies a *BMP* dependent timer in these anterior regions too and indicates that, while the timer includes *Hox* TC, it also exceeds it. In mouse, no *BMP* or anti *BMP* dependence has yet been shown but stabilization of a series of unstable nascent A-P identities in primitive streak cells by signals from a stable organizer derived cell population has been shown (Wymeersch et al., 2018). This suggests the same mechanism as in the other vertebrates where anti *BMP* signals from the organizer stabilize nascent *Hox* codes in *BMP* rich pluripotent ventral cells (in NOM mesoderm (Xenopus/anamniote) or in posterior primitive streak (chicken/amniote).

Conclusion: Regulation of Hox collinearities by BMP-anti BMP occurs generally in vertebrates and is central in a core collinearity mechanism. This regulation is central in one of the two models (dur). It is thought, together with the collinear opening of chromatin and Hox-Hox interactions to comprise the basic integral core time-space translation mechanism for collinearity (dur). It is not mentioned in the other model (dedu).

## What Is Involved in the Molecular Mechanisms of *Hox* Collinearity and Axial Patterning?

### Collinear Chromatin Opening?

There is evidence that a *cis-*acting mechanism of this nature is involved. It is regulated by TAD’s (topologically associating domains), each containing multiple enhancers, there being two TAD’s: one at each end of each *Hox* cluster so far studied (Deschamps and Duboule, 2017). This *cis-*acting mechanism appears to involve changes in chromatin architecture, with removal of inhibitory marks on chromatin histones and addition of activating ones (Deschamps and Duboule, 2017). Being *cis-*acting, this type of process alone cannot account for the synchronization and coordination of different *Hox* clusters and of different cells that make collinearities multiscalar and observable at the multicellular level of the embryo (Durston, 2018).

Conclusion: Collinear chromatin opening (dedu) is generally important. It could account for the connection between collinear Hox gene expression and corresponding genomic position. It is presumably part of the core mechanism. This is inherently a cis-acting, single cell mechanism that requires intercellular signaling to synchronize and coordinate it. It is acknowledged in both models. Notably, if chromatin opening is to be visible and detectable in multicellular situations, which it is, this intercellular signaling always needs to be available and active. An “open by business” chromatin model is indicated.

### Is a *Hox-Hox* Interaction (PI) Involved?

Loss of function (LOF) and gain of function (GOF) experiments for *Hox* genes point to involvement of a *Hox* function in collinearity. Strikingly, antisense *Hox* RNA treatments of synchronized temporally collinear pluripotent *NT2/D1* human *EC* cells caused cascade LOF phenotypes where LOF for *Hoxb1* or *Hoxb3* blocked expression of all later expressed more 5′ *Hox* genes in all 4 clusters (Faiella et al., 1994). This indicated that a *Hox-Hox* interaction, posterior induction (PI), where more anterior *Hox* genes induce their posterior neighbors, is involved in TC. In *Xenopus* embryos, comparable SC phenotypes were obtained, emphasizing the connection between temporal and SC. LOF for all 3 *Xenopus Hox1* genes deleted or strongly reduced expression of all more 5′ posterior *Hox* genes in all 4 clusters (McNulty et al., 2005). LOF for *Hoxc6* deleted or strongly reduced expression of all more 5′ posterior *Hox* genes in all 4 clusters (Zhu et al., 2017b). In addition, *Hox1* LOF enhanced expression of the immediately anterior zonal marker *Gbx2* and *Hoxc6* LOF enhanced expression of the immediately more anterior *Hox* genes *Hoxb4*, and *Hoxb5*. The above results emphasize that *Hox* LOF acts in trans. The LOF results were obtained, like the *NT2/D1* LOF results, using antisense technology (in this case morpholinos) and repeats using other approaches (e.g., CRISPR) would be desirable but the high specificity of the phenotypes obtained leaves no doubt as to the specificity of this approach. In addition to these LOF results, GOF experiments (ectopic expression by microinjection of mRNA) with *Hoxd1*, *Hoxb4*, *Hoxa7*, *Hoxb9* initiated posterior partial axes in ventralised (*Hox* free) and wild type *Xenopus* embryos with the axis starting at the ectopically expressed *Hox* gene in each case (Hooiveld et al., 1999; Zhu et al., 2017a). Again, these are very specific phenotypes that indicate a specific result. The facts that these LOF and GOF phenotypes involve effects on all 13 paralog groups and all 4 *Hox* clusters and that these effects were induced by 8 different manipulations of 7 different *Hox* genes leave no doubt that *Hox* interactions have a general role in collinearity. This role is obviously *trans* acting between *Hox* clusters, and the fact that *Hox* GOF can induce a full *Hox* axis with defined coordinated zones indicates that the PI interaction (involved here) acts non-cell autonomously. It is also obvious that for PI to be able to work, it needs to be restricted to acting directly only on near posterior neighboring *Hox* genes. This was tested for one case: *Hoxb4*, acting on *Hoxb5*, *Hoxb7*, *Hoxb9*. In this case, *Hoxb5* was indeed the only direct target. *Hoxb7, Hoxb9* were indirect targets (Hooiveld et al., 1999). *Hox* response elements that could mediate a PI like interaction and Au regulating response elements (below) have been identified in different *Hox* genes. It is possible that the restriction of PI to close posterior *Hox* neighbors reflects collinear chromatin opening. *Conclusion: The role of PI is proposed only in one model (dur). It is not mentioned in the other (dedu). PI and the other *Hox-Hox* interactions are proposed to be part of the basic core mechanism for collinearity (dur).*

### Are Other *Hox-Hox* Interactions Involved?

Besides PI, other *Hox-Hox* interactions are involved in collinearity. Following the onset of PI in *Xenopus* (which is already active with expression of the first *Hox* gene early in gastrulation), a second interaction starts later. Posterior *Hox* genes begin to repress expression of more anterior ones (Zhu et al., 2017a). This interaction: posterior dominance (PD) is probably required for stabilizing *Hox* zones and thus for the switch from TC to SC. It starts around stages 12–15 (end gastrula to mid neurula) in *Xenopus*. This interaction is imposed by *Hox* genes and also by the *Hox* associated *miRNa*′*s*: *Mir10* and *Mir196* (Woltering and Durston, 2008; Yekta et al., 2008). In all cases of *Hox* PD examined by us and in the known cases of *miRNA* imposed PD, this interaction involves regulation at the *Hox* mRNA level as well as regulation of *Hox* function. In this respect, this interaction differs from (being broader than) the similar *Hox*-*Hox* interaction: posterior prevalence, proposed previously by D. Duboule, which, like *Drosophila* “phenotypic suppression,” was proposed to be restricted to action at the posttranslational functional level (Duboule, 1991; Duboule and Morata, 1994). Beside PI and PD, there is a third interaction: autoregulation (A) whereby for example, mesodermal *Hox* identities are copied over to overlying neurectoderm (Bardine et al., 2014). This interaction is clearly non-cell autonomous in this particular situation.

Conclusion: These interactions feature in the dur model. Dedu mention and therefore presumably accept only the old studies on “posterior prevalence” and PD like interactions imposed by Hox13.

### Is *Hox* Controlled Cell Ingression Involved?

Experiments in the chicken embryo showed that ectopically expressing a *Hox* gene in a primitive streak cell determines time of ingression and therefore migration of this cell during gastrulation. Ectopic expression of an anteriorly expressed *Hox* gene causes early ingression taking the cell to an anterior position at the end of gastrulation. A more posteriorly expressed *Hox* gene causes later ingression, leading to the correct, more posterior position, being reached later in gastrulation (Iimura and Pourquie, 2006; Denans et al., 2015). This no doubt contributes to the patterning process. This process is putatively important in amniotes like chicken, where cells ingress individually during gastrulation. It may be less important in anamniotes like frog, where mesoderm cells involute as a sheet during gastrulation.

Conclusion: This movement control likely contributes to axial patterning. It alone is not sufficient to account for the transition from TC to SC (this feature is regrettably, not discussed in either model).

General conclusion: There is clear evidence for the roles of collinear chromatin opening (dedu, accepted by both models), for the roles of the PI. PD and A Hox-Hox interactions (dur, not mentioned by dedu) and of Hox controlled cell migration during gastrulation (regrettably, discussed by neither), in collinearity. BMP-anti BMP, Collinear chromatin opening and Collinear Hox-Hox interactions together appear to be main components of a basic integral core collinearity mechanism that applies for all Hox genes and interacts with external signaling pathways that each act only on a part of the 3′–5′Hox sequence (dur, and see below).

## How Is *Hox* Collinearity Coordinated/Synchronized at the Multicellular Level?

*Cis-*acting or cell localized processes like collinear chromatin opening and possibly like *Hox-Hox* interactions need to be connected, synchronized and coordinated via intercellular signaling to be effective and to be detectable at the multicellular level (Durston, 2018). RNA seq. analysis of single limb cells (Fabre et al., 2018) reveals considerable variation in *Hox* expression between individual cells but there is clearly enough coordination to generate the global collinearity phenomena that are observed. How is this coordination achieved?

### External Morphogen Signaling Pathways?

Both the dedu and dur models propose that an A-P series of external signaling pathways synchronize TC at different times, corresponding to different A-P levels. Dedu mention three morphogens: Wnt, (3/3A in mouse), Cdx, Gdf11, working at an A-P series of levels (Deschamps and Duboule, 2017). Dur proposes roles for these and for other morphogens too (Durston, 2015, 2019c). The idea is that these three pathways synchronize TC at specific times/A-P levels. Interestingly, the *Wnt* and *Cdx* pathways are known to have response elements acting at approximately the right levels in the axial sequence of *Hox* genes to do this (Deschamps and Duboule, 2017). *Wnt* responsive elements act early in the 3′part of the *Hox* sequence. Some regulate *Hoxa1* directly (Neijts et al., 2016). *Cdx* elements act later in the middle of the axis following *Wnt* induction of *Cdx* (Neijts et al., 2017).

*Wnt8* (the Xenopus functional equivalent of murine *Wnt3*) was found to induce only *Hox1* paralog (*Hoxa1*, *Hoxb1*, *Hoxd1*) directly. It induced it’s other *Hox* targets that were detected: *Hoxb4*, *Hoxd4*, *Hoxc6*, *Hoxa7*, *Hoxc8*, indirectly (In der Rieden et al., 2010). Expression of the earliest, most anteriorly expressed *Xenopus Hox* gene induced by *Cdx: Hoxc6*, was also found to be required for the expression of all more 5′ posterior *Xenopus Hox* genes (Pownall et al., 1996; Zhu et al., 2017b). A member of *Hoxc6’s* immediately anterior neighboring *Hox* paralog group: *Hoxa5* was also found to *be* induced by *Cdx* loss if function (i.e., to be repressed by *Cdx)* (Neijts et al., 2017). This recalls the induction of *Hox5* genes by *Hoxc6* LOF (see above). Perhaps *Hox1* genes and *Hoxc6* are the only essential direct *Wnt* and *Cdx* targets respectively for TC and perhaps only the first *Hox* gene expressed in each axial domain is the essential direct morphogen target, the others being capable of being induced indirectly via the PI *Hox-Hox* interaction. A similar conclusion is indicated for action of a third morphogen class: retinoids (Durston, 2019c). Dur noticed that the axial positions where members of the 3′–5′ axial sequence of morphogen signaling pathways initiate their action correspond exactly to the decision points between sequential anatomical domains on the A-P axis. *Wnt* acts at the boundary between anterior and posterior head; corresponding to posterior/later initiation of the rhombencephalon and of occipital somites; *Cdx* acts at the boundary between neck and thorax, corresponding to termination of rhombencephalon and cervical somites and initiation of the spinal cord and thoracic somites (Durston, 2019c). He suggested that these signaling pathways are external to the integral core collinearity mechanism and that their function is to regulate domain switches by being superimposed on it, in each case upregulating the *Hox* gene or paralog group immediately after a decision point in an extra level of control (Durston, 2015, 2019c). In contrast, dedu assume that these external morphogen signaling pathways are the only means of intercellular communication.

### BMP and Non-cell Autonomous *Hox-Hox* Interactions?

In addition to the above A-P morphogens, *BMP*-anti-*BMP* appears to play a general role in mediating the basic integral core collinearity mechanism (see above). In addition, chromatin opening and *Hox* interactions, including PI and A, which, like *BMP*-anti *BMP*, act through the whole *Hox* sequence, appear to be part of this core mechanism. These interactions appear, interestingly, to be non-cell-autonomous. Their intercellular action may enable non-cell autonomy of temporal and spatial *Hox* collinearities in the core mechanism. Perhaps, collinear PI also causes or relies on collinear chromatin opening. Non-cell autonomy may be mediated by *Hox* genes activating and being activated by traditional signaling pathways (like *BMP*). It may alternatively be mediated by *Hox* proteins being transported directly from cell to cell (Dupont et al., 2007). It is also possible that ‘*Hox-Hox* interactions are passed from cell to cell due to cell lineage inheritance (below). Note that none of these features are found in dedu, which assumes that external morphogen signaling pathways are the only relevant means of intercellular communication.

Conclusion: Coordination and synchronization at the multicellular level is key to collinearity. It is what makes it detectable. That this is mediated purely by an early-anterior to late-posterior sequence of morphogens, external to the collinearity mechanism (dedu) is perhaps unattractive. On the other hand, that these morphogens, which are undeniably involved, feed into and influence an integral basic core functional collinearity mechanism, and that they define axial domains (dur) seems much more likely.

## What Is the Embryology of Axial Patterning?

There are two main tissues in the vertebrate embryo that carry the A-P axial pattern: First: axial mesoderm: that starts out as involuting/ingressing NOM/primitive streak in the gastrula and goes on to become paraxial/presomitic mesoderm post gastrulation. Second: axial neurectoderm: the precursor of the central nervous system. There are two ideas about how these patterns arise and how they are connected.

### Activation-Transformation?

The classical idea comes from Amphibian embryology. It says that A-P axial levels are first specified in axial mesoderm (we would suggest by time-space translation following an interaction between NOM or primitive streak and the embryo’s organizer). These mesodermal A-P levels are then copied over to neurectoderm (which lies adjacent to axial mesoderm in the embryo). This mechanism (activation-transformation) was discovered in Amphibia (anamniote) but was confirmed and elaborated in Chick (amniote) (Mangold, 1933; Nieuwkoop, 1952; Stern, 2005; Bardine et al., 2014). This idea is well established and based on much experimental evidence, with explants, recombinates, lineage analysis etc. The evidence is particularly well known in Amphibia but has also been demonstrated in chick. It surely also applies in mouse (Metzis et al., 2018).

### Cell Lineage?

Second, there are recent exciting findings showing that the embryonic precursors that develop the axial pattern are precursors for mesodermal as well as neural tissue. These pluripotent precursors (NMP’s) are postulated to acquire A-P positional information already at their pluripotent stage, then to divide and grow and, at a certain point in time to generate purely mesodermal and purely neural progeny. The ideas for this alternative were developed in mouse, by single cell lineage tracing and other approaches (Tzouanacou et al., 2009; Wymeersch et al., 2016; Metzis et al., 2018). This idea is backed by substantial evidence. It is very attractive because it potentially provides a convenient way to pass on positional information from cell to cell, in parallel to mesoderm and neurectoderm without intercellular signaling being involved, simply by cell division. This would enable cell autonomous patterning processes like chromatin opening and any cell autonomous *Hox-Hox* interactions to be passed from cell to cell. This general embryology situation is thus complex, with main questions unsolved. It appears that different mechanisms: activation-transformation and pluripotent cell lineage are involved in patterning axial mesoderm and neurectoderm in vertebrate embryos. These different modes may possibly operate at different stages of the patterning process and may have different importance in different vertebrates. Please note that intercellular signaling is still nonetheless essential to synchronize and coordinate collinearities and make them observable.

Conclusion: Our two models each regrettably use only one of the two ideas that have been proposed to underly vertebrate axial differentiation and patterning. Namely, intercellular signaling (dur) and pluripotent cell lineage (dedu). The embryology is unattractively complex at this time.

## The Precision of Axial Patterning: How Could This Be Explained?

### What Are the Aspects of Precision?

Could the mechanisms above explain axial patterning with the necessary precision? Some features of these mechanisms are worrying with regard to precision. For example, if the timing of a cell autonomous function, like chromatin opening, is synchronized at only every fourth to sixth *Hox* gene by external, extracellular A-P signals like *Wnt*, or *FGF*, are such extracellular signals external to and independent of, the integrated core collinearity mechanism, with no feedback from it? These aspects require investigation and make the potential role of non-cell autonomous *Hox-Hox* interactions (which could potentially provide very close control) interesting. Another aspect that provides food for thought is the question of how signals are delivered. Is this a question of a morphogen concentration exceeding a threshold (a typical analog signal). Could a signal like this time TC precisely enough in a sequence of *Hox* genes?

### Is High Precision Timing Involved?

On the other hand, there is a different, high precision timing device active in the same tissues as *Hox* TC that may possibly drive it. This is: the somitogenesis clock; a relatively high frequency oscillator that has a rather constant stable period, presumably due to having limit cycle characteristics. This could measure time with precision; like a quality Swiss watch, by counting the number of elapsed oscillator cycles (ticks of the watch). This timer runs in exactly the same tissues and over exactly the same time course as *Hox* TC (Palmeirim et al., 1997; Jouve et al., 2002; Peres et al., 2006; Riedel-Kruse et al., 2007). It is also coupled to collinearity in the way expected if it drives it: different oscillator cycle numbers, generate differently numbered somite boundaries, corresponding to different *Hox* anterior expression boundaries. LOF for the somitogenesis clock disrupts *Hox* axial patterning (Zakany et al., 2001; Peres et al., 2006). A recent theoretical model (Kudlicki, 2019) has devised a digital molecular mechanism whereby somitogenesis clock cycle No. could be counted, allowing elapsed time to be translated to A-P position.

Conclusion: Precision is possibly a problem. An integrated timing mechanism would help. Involvement of the highly precise somitogenesis clock as a driver and timing by counting oscillation cycles would introduce a much higher level of precision.

## What Do We Know?

**FIGURE 2:**
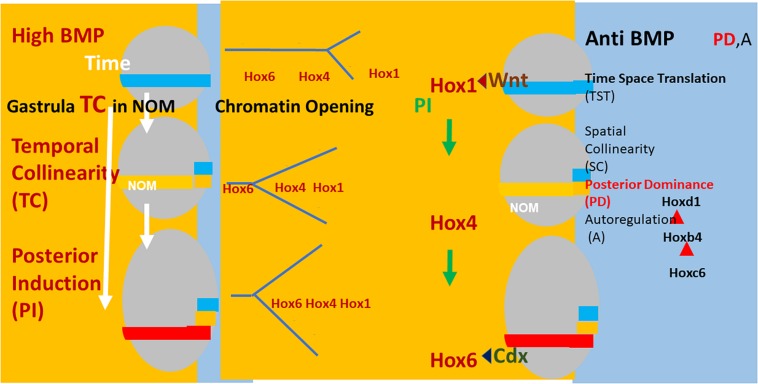
**Left:** (Xenopus) embryos (gray ovals) at sequential stages in gastrulation. The NOM mesoderm (horizontal colored stripe) runs from ventral to near dorsal. I show some of the successive stages of *Hox* expression in NOM. It is first blue (Hoxd1 is the latest/most posterior gene expressed at this stage). Then yellow (Hoxb4 is the latest/most posterior gene expressed at this stage). Then red (Hoxc6 latest/most posterior expressed). These are three stages in the first part of the NOM temporally collinear *Hox* sequence. The yellow background to the figures shows that TC happens in availability of a high BMP concentration, which is available in most of the **(left)** ventrolateral part (V) of the embryos (as shown). Under these conditions, collinear opening of chromatin and the *Hox-Hox* interaction PI also occur as do Wnt and Cdx inputs into the Hox1 genes and Hoxc6, respectively. These activities all have a yellow background, indicating that they require high BMP conditions. A thin segment at the **(right)** dorsal: D side of the embryo has a blue background (shown fully only for the identical embryos at the right hand side of the figure). This represents anti BMP, which is available in the dorsal side of the embryo (D) only. Under these conditions, successive blocks of cells are frozen at each successive *Hox* code and these blocks stack up to make an axis. This process involves making mesodermal and neural layers of spatially collinear tissue (not shown). It correlates with and presumably involves two late *Hox-Hox* interactions, posterior dominance, whereby posterior *Hox* genes inhibit function of and repress expression of more anterior *Hox* genes and Autoregulation, whereby mesodermal Hox expression is copied over non-cell autonomously to neural tissue.

Figure 2 and Tables 1, 2 summarize the present knowledge.

## Discussion: Best Guess Hypothesis and Future Prospects. How Might the Vertebrate Axial Patterning Mechanism Look?

### Best Guess Hypothesis

(a) *Hox TC exists.* The best evidence comes from *in situ* hybridization analysis, which enables detecting onset of *Hox* expression at the appropriate stage in the appropriate tissue. TC appears to be near perfect.
(b) *TC leads to SC.* Evidence from *BMP*/anti *BMP* regulation of *Hox* collinearity and patterning in *Xenopus.* Only in the dur model but the evidence is strong.
(c) *BMP/anti BMP regulation of axial patterning and collinearity is general in vertebrates.* Demonstrated in *Xenopus*, chicken, zebrafish. Almost definite in mouse. Strong evidence.
(d) *Collinearities (TC and SC) are mediated by a basic integral core mechanism, involving; BMP/anti BMP; collinear *Hox* chromatin opening; collinear *Hox-Hox* interactions (PI, PD) and A; *Hox* regulated cell ingression.* This core mechanism is proposed only in the dur model, except for *Hox* regulated cell ingression [Pourquie and colleagues (Iimura and Pourquie, 2006; Denans et al., 2015)] and collinear chromatin opening and posterior prevalence (dedu). The evidence for it is strong.
(e) Because some components of the core collinearity mechanism are cis acting/cell localized, intercellular communication is needed to synchronize/coordinate them in the multicellular embryo.

### Two Types of Communication Are Proposed

(i) *A-P morphogen signalling pathways, external to the basic integral core collinearity mechanism. Eg: Wnt, Cdx, Gdf11.* These regulate collinearity over particular stretches of the axis. There is evidence that these stretches are axial morphological domains and that the morphogen pathways each serve to co-upregulate expression of the first (most anterior) *Hox* gene in the domain after a particular “decision point” and that this regulates the other *Hox* genes via PI. Please note that, for two of the three best characterized “decision points,” (retinoids/*Hox1* and *Cdx/Hox6*), the axial determinant immediately anterior to the decision point is also downregulated by the morphogen, as if this drives the *Hox-Hox* interaction PD. I note that there are some pathway response elements that directly regulate *Hox* genes other than the first *Hox* gene after each decision point (e.g., Charite et al., 1998; Schyr et al., 2012). These are evidently not essential for the morphogen pathway regulation of *Hox* collinearity.
(ii) *Communication as part of the core mechanism.* The most important here is non-cell autonomous *Hox-Hox* interactions (PI, PD, A). These mediate collinearities and participate in mediating domain switches. Apart from these, *BMP/anti BMP* plays a permissive role, in determining which aspect of the collinearity mechanism is enabled.

### The Nature of the Embryology

(i) *Classical studies revealed that cell interactions* Are *central.* Particularly the activation-transformation interactions that mediate transfer of patterning information from axial mesoderm to axial neural tissue. These conclusions are backed by abundant experimental evidence.
(ii) *Recent studies in mouse revealed that common neural- mesodermal precursors develop A-P identities before these cell types diverge.* This exciting conclusion is backed by solid experimental evidence. It raises the exciting possibility that A-P identities can be passed from cell to cell without intercellular signalling. The role of common precursors (NMP’s) needs to be defined more precisely.
(iii) *Precision.* The requirements for precision are unclear. If required, very precise timing could be imposed via the somitogenesis clock. The role of the somitogenesis clock is unclear.

### Future Prospects

#### Perspectives for Medicine

*Can These Insights Be Used in Connection with Stem Cells?* The mechanism above is an important part of the body plan program that generates the diversity of cell types and organs that make a vertebrate. Investigations by Faiella et al. (1994) already demonstrated a long time ago that part of this mechanism can operate in a pluripotent cell line. The study by Faiella et al. (1994) also first demonstrated the PI *Hox-Hox* interaction. Recent pubications (Seifert et al., 2015; Smith et al., 2019) have emphasized that *Hox* genes are important in regulating ES stem cell directionality. The cells involved in the embryo clearly include pluripotent stem cells too. With the diversity of ES cells now available, it will be important to determine whether this *Hox* mechanism can be used to generate and further new stem cell applications. It should also have perspectives for *in vitro* organoid culture. I hope someone will explore this. I would do it myself if I weren’t too old.

#### Future Investigation of the Nature of the Mechanism

The bones of the axial patterning/collinearity mechanism are now perhaps becoming clear. There are, however, key questions that still need to be settled definitively.

(i) *Does Hox TC actually exist?* Two recent publications questioned whether *Hox* TC actually exists (Kondo et al., 2017, 2019). I have presented the arguments that it does and that it is of central importance (Durston, 2019a,b and see above). This question needs to be settled urgently and definitively.
(ii) *What is the nature of the timer? Hox* TC drives the timing and spatial sequence of axial patterning. But is TC itself the driver or is it in turn driven by something else? Is it itself precise enough to drive a developmental program? This is an important question. The degree of precision required needs to be determined. There is a second very precise time-space translation mechanism active in the early embryo, in the same tissues and with the same timing as *Hox* TC. This mechanism (the somitogenesis clock) is presumably precise because it is based on (many ticks of) a relatively high frequency oscillator (the limit cycle characteristics of which should ensure stability) and it is known to be able to drive *Hox* TC (Peres et al., 2006). TC, however, also feeds back to drive it (McNulty et al., 2005). These two TST mechanisms are thus clearly connected. What drives what and when and where?
(iii) *What is the nature of Hox-Hox interactions?* The mechanism for generating *Hox* TC and translating it to a spatially collinear pattern is complex. Multiple collinear *Hox-Hox* interactions appear to be involved. TC appears to require PI. PI was deduced from cascade phenotypes in *Xenopus* and in NT2/D1 cells which were all obtained using either ectopic expression (GOF) or antisense technology (morpholinos or regular antisense oligonucleotides; LOF). These phenotypes appeared very specific and not artifactual because each generated expression of a very specific sequence of *Hox* genes. However, it would be instructive to see what kinds of *Hox* expression phenotypes other standard gene manipulation approaches (like ectopic expression in mouse, homologous recombination in mouse, CRISPR) give. This is so far largely unknown. In addition, it is absolutely necessary to identify and catalog enhancers and any other regulatory motifs that mediate these interactions.
(iv) *What are the roles of morphogens?* There are various morphogens that are thought to be involved in setting up the A-P axis. Their roles in relation to the timing mechanism considered here have been discussed above and elsewhere (e g., Durston, 2019c). However, this aspect deserves much further attention. There is lots more to be done.

## Author Contributions

The author confirms being the sole contributor of this work and has approved it for publication.

## Conflict of Interest

The author declares that the research was conducted in the absence of any commercial or financial relationships that could be construed as a potential conflict of interest.
